# Evaluation of Corrosion Performance of AZ31 Mg Alloy in Physiological and Highly Corrosive Solutions

**DOI:** 10.1021/acsabm.3c01169

**Published:** 2024-02-27

**Authors:** Berzah Yavuzyegit, Aikaterina Karali, Arianna De Mori, Nigel Smith, Sergey Usov, Pavel Shashkov, Roxane Bonithon, Gordon Blunn

**Affiliations:** † School of Pharmacy and Biomedical Sciences, Faculty of Science and Health, University of Portsmouth, St Michael’s Building, White Swan Road, Portsmouth PO1 2DT, U.K.; ‡ Mechanical Engineering Department, Recep Tayyip Erdogan University, Rize 53100, Turkey; § School of Mechanical & Design Engineering Faculty of Technology, University of Portsmouth, Anglesea Building, Anglesea Road, Portsmouth PO1 3DJ, U.K.; ∥ BioCera Medical Limited, 3b Homefield Road, Haverhill CB9 8QP, Suffolk, U.K.

**Keywords:** corrosion media, biodegradable implants, biomaterials, corrosion inhibitor, surface modification

## Abstract

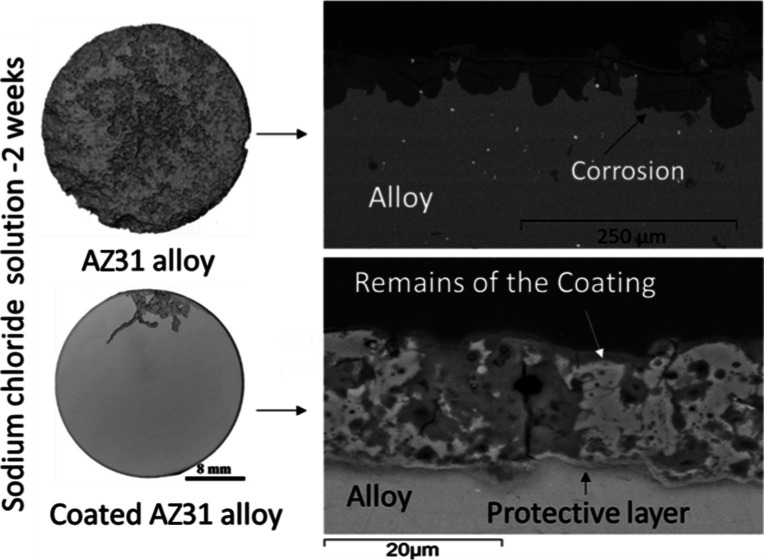

Resorbable Mg and Mg alloys have gained significant interest as promising biomedical materials. However, corrosion of these alloys can lead to premature reduction in their mechanical properties, and therefore their corrosion rate needs to be controlled. The aim of this study is to select an appropriate environment where the effects of coatings on the corrosion rate of the underlying Mg alloy can be discerned and measured in a relatively short time period. The corrosion resistance of uncoated AZ31 alloy in different solutions [Hank’s Balanced Salt Solution, 1× phosphate buffered solution (PBS), 4× PBS, 0.9%, 3.5%, and 5 M sodium chloride (NaCl)] was determined by measuring the weight loss over a 2 week period. Upon exposure to physiological solutions, the uncoated AZ31 alloys exhibited a variable weight increase of 0.4 ± 0.4%. 3.5% and 5 M NaCl solutions led to 0.27 and 9.7 mm/year corrosion rates, respectively, where the compositions of corrosion products from AZ31 in all saline solutions were similar. However, the corrosion of the AZ31 alloy when coated by electrochemical oxidation with two phosphate coatings, one containing fluorine (PF) and another containing both fluorine and silica (PFS), showed 0.3 and 0.25 mm/year corrosion rates, respectively. This is more than 30 times lower than that of the uncoated alloy (7.8 mm/year), making them promising candidates for corrosion protection in severe corrosive environments. Cross-sections of the samples showed that the coatings protected the alloy from corrosion by preventing access of saline to the alloy surface, and this was further reinforced by corrosion products from both the alloy and the coatings forming an additional barrier. The information in this paper provides a methodology for evaluating the effects of coatings on the rate of corrosion of magnesium alloys.

## 1 Introduction

Mg and Mg alloys have gained significant interest as a promising biomedical material for surgical fixation of musculoskeletal devices such as bone screws and bone plates and for intraluminal stents in cardiovascular applications.^1,2^ Their biodegradable and biocompatible nature eliminates the need for surgical removal once their purpose is fulfilled.^3^ These materials exhibit strength and density closer to those of cortical bone than any other metallic biomaterials. However, their susceptibility to corrosion, particularly in chloride-rich saline environments, compromises their structural integrity and performance.^4,5^ Additionally, the rapid emission of hydrogen gas and the subsequent gas bubble formation within the tissue during corrosion restrict the widespread use of Mg alloys as biomaterials. Therefore, enhancing the corrosion resistance of Mg alloys by using coatings is crucial to fully realize their potential in biomedical applications.^6^

Corrosion tests serve as a cost-effective in vitro alternative to expensive in vivo animal trials during the initial stages of alloy development for biomedical applications. To enhance the corrosion testing of Mg alloys and replicate the physiological environment of the human body, different types of solutions have been utilized and developed. Mei et al.^7^ categorized commonly used corrosive media; the most prevalent media for corrosion tests include 0.9% NaCl solution (saline)^8,9^ and 3.5% NaCl solution;^10^ solutions employed in biological research and biomedical applications such as phosphate-buffered saline solution (PBS);^11^ and simulated body fluids containing inorganic ions found in body fluid in addition to NaCl, Hank’s balanced salt solution (HBSS),^12−14^ and Earle’s balanced salt solution (EBSS), which are both constituents of cell culture media utilized for in vitro cell biology research,^15^ and are protein-containing media.^16^

Mg undergoes oxidative degradation when it is exposed to water, forming Mg(OH)_2_ and H_2_. The main chemical reactions are as follows^1^
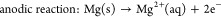
1
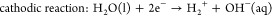
2
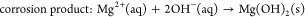
3

Mg dissolution, accompanied by hydrogen evolution, results in an elevation of the OH^–^ ion concentration within the solution. Reaction (3) forms a protection layer of Mg(OH)_2_, which has a low solubility in water and is deposited on the thin layer of MgO that forms on the surface of the Mg substrate prior to immersion. However, in aqueous solutions containing the chlorine ion (Cl^–^) at concentrations higher than 30 mmol/L, this layer reacts with Cl^–^ to form highly soluble MgCl_2_. The released OH^–^ increases the pH of the solution. As the chloride concentration in body fluids is around 150 mmol/L, the degradation of Mg alloys is associated with the generation of hydrogen and the buildup of corrosion products.^3,17^

Previous studies have investigated the corrosion behavior of different Mg alloys and coatings in various solutions, providing insights into the effects of alloy composition, surface finish, microstructure, grain size, solution compositions, and test duration that influence corrosion rates. Tkacz et al.^4^ examined the corrosion behavior of two Al–Zn–Mg alloys, namely AZ31 and AZ61, in HBSS. The study focused on analyzing the influence of phase distribution, surface finish, and microstructure of the alloys. Electrochemical impedance spectroscopy showed that AZ31 exhibited a higher degree of corrosion sensitivity compared to AZ61, while the results from potentiodynamic tests showed the opposite trend. Surface finish had a minimal impact on AZ31, while it had no significant effect on AZ61 corrosion, as evident from the potentiodynamic tests. In another study, Zhu et al.^1^ examined the corrosion behavior and formation of Mg(OH)_2_ on the AZ31 Mg alloy in HBSS. The researchers concluded that the corrosion rate of AZ31 significantly decreases as the thickness of the coating increases. Alvarez-Lopez et al.^18^ examined the corrosion resistance of AZ31 Mg alloy when exposed to a 0.8% NaCl solution and PBS, focusing on the influence of grain size and test duration. Interestingly, they discovered that the corrosion rate showed no significant variation based on the grain size. However, during the initial stages of testing, the corrosion rate of the alloy in PBS was higher compared with the 0.8% NaCl solution. As the test duration increased, the corrosion rate of PBS decreased below that of the 0.8% NaCl solution. Kim et al.^11^ studied the corrosion behavior of AZ31 Mg alloys with ultrafine grain in PBS, and they found that the corrosion rate significantly decreases as the grain size reduces. Furthermore, Han et al.^19^ conducted a comprehensive investigation into the corrosion characteristics of strain-hardened AZ31B Mg alloy under circulating flow conditions in various environments, including saline, PBS, and simulated body fluid. Their findings revealed that the corrosion rate of the alloy was the highest when immersed in saline. Altun and Sen^20^ studied the effect of Cl^–^ ion concentration and pH on the corrosion behavior of AZ63 Mg alloy immersed in NaCl solutions with varying concentrations and pH values. They concluded that the corrosion rate increases with increasing Cl^–^ ion concentration and decreasing pH.

A variety of surface treatments have been offered to reduce the corrosion rate of Mg alloys. They include chemical conversion, biomimetic deposition, sol–gel, anodizing, and plasma electrolytic oxidation (PEO).^21,22^ In our study, we investigated a novel surface treatment by electrochemical oxidation (ECO) in phosphate-based electrolytes. ECO treatment combines oxidation of the substrate in an electrolytic tank by high-frequency electrical pulses with elementary codeposition of material from the electrolyte, resulting in a surface coating that presents polycrystalline Mg oxide enriched with phosphate, fluoride, and silicate ions. ECO treatment was reported to have superior mechanical properties compared to state-of-the art anodizing and PEO on Mg.^23^

It is important to understand the corrosion behavior of Mg alloys and the effects that coatings have on their corrosion rates as this will affect their applications in a physiological environment. This study aims to select an appropriate environment in which the effects of coatings on the corrosion rate of underlying magnesium alloy can be discerned and measured in a relatively short time. The objectives of this study are to investigate the corrosion behavior of AZ31 Mg alloy under different solutions, namely HBSS, PBS, 4× PBS, and NaCl, with varying Cl^–^ concentrations in order to develop a quick and effective testing method that will be able to discern, differentiate, and predict the effect of different coatings on the corrosion of this alloy when used for biomedical applications. These findings will help in the development of effective corrosion mitigation strategies in physiological applications.

## 2 Material and Methods

### 2.1 Sample Preparation

Five mm thick discs were cut from a 25 mm diameter bar of AZ31 Mg alloy (Mg–Al 3 wt %, Zn 1 wt %) (Goodfellow Cambridge Limited—UK, Ermine Business Park. Huntingdon, UK). The surface area of each disc was 13.74 cm^2^. The Mg alloy samples were treated with either a phosphate and fluoride electrolyte (PF coating) or phosphate, silicate, and fluoride electrolyte (PFS coating) in 15 μm thicknesses and were provided by BioCera Medical Ltd. (Haverhill, England).

The applied surfacing technology is a version of soft-sparking PEO developed and patented by Biocera Medical Ltd. (PCT publication WO 2020049299). This technology, denoted as ECO (electrochemical oxidation), enables a reduction in the sparking (discharge) effects inherent to conventional PEO coatings. It leads to a more compact and denser layer, with roughness controlled by the process’s electrical parameters. The ECO process was conducted by the application of positive and negative electrical pulses of +500 and −150 V accordingly at a pulse repetition frequency of 1 kHz. A programmable power supply maintained potentiostatic mode for positive pulses and galvanostatic mode for negative pulses, which avoided breakdown discharge sparking during the growing layer thicknesses and resulted in the formation of a controlled nanocrystalline ceramic structure.

The PF and PFS electrolytes represented water-based solutions of phosphates, fluorides, and silicates of alkali metals in low concentrations, typically below a concentration of 5 g/L. Electrical conductivity of both electrolytes was maintained at a constant operational level of 25 mS by adjusting the concentration of potassium hydroxide (KOH) in the electrolyte solution. The presence of phosphate in both electrolyte systems supports the biocompatibility of the formed nanoceramic layer and was instrumental in increasing its hardness. Fluoride ions function to compact the layer and potentially enhance antibacterial properties.^24^ Silicate is known to be beneficial for early bone formation, osseointegration, and accelerated layer growth (build-up).^25^

### 2.2 Corrosion Tests

#### 2.2.1 Immersion Tests of Uncoated AZ31 Magnesium Alloy

The 7 uncoated AZ31 Mg alloy samples were used to determine the appropriate testing media so that the coated alloy samples could be differentiated. A number of solutions were used as test media: (1) HBSS (Gibco, Thermo Fisher, USA) at room temperature (RT), (2) 1× PBS at RT, (3) 4× PBS at 37 °C, (4) 0.9 wt % NaCl solution (9 g/L) at 37 °C, (5) 3.5 wt % NaCl solution (35 g/L) at 37 °C, (6) 5 M NaCl solution (292 g/L) at 37 °C, and (7) 5 M NaCl solution (292 g/L) by temporarily removing them from the corrosion solution at 37 °C (interrupted 5 M NaCl solution). HBSS solution contains 0.35 g/L NaHCO_3_, 1 g/L glucose, 8 g/L NaCl, 0.4 g/L KCl, 0.04 g/L Na_2_HPO_4_, 0.06 g/L KH_2_PO_4_, 0.20 g/L MgSO_4_·7H_2_O, and 0.14 g/L CaCl_2_. PBS solution contained 8 g/L NaCl, 0.2 g/L KCl, 2.89 g/L Na_2_HPO_4_·12H_2_O, and 0.2 g/L KH_2_PO_4_.

Before the tests, each sample was ultrasonically cleaned (XUBA3, Grant Instruments, Cambridgeshire, UK) in ethanol (99%+) for 15 min and dried in air for 30 min. The weight of the discs was taken using a precision balance (Sartorius balance, Göttingen, Germany), and then the discs were suspended vertically in 100 mL solutions for 14 days at RT or 37 °C, with the fluid being gently swirled using an orbital shaker (Orbi-Shaker, Benchmark, USA) set to 30 rpm. For the interrupted tests, the samples were taken after 24 or 48 h, ultrasonically cleaned in ethanol for 15 min to remove the corrosion products, dried in air for 30 min, weighed, and then suspended back into fresh media.

The corrosion rate was calculated by the following equation
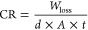
4where *W*_loss_ is weight loss, *d* is density, *A* is the total area of the sample subjected to corrosion, and *t* is exposure time of the sample to the corrosive media.

The pH of the solutions was measured by taking a sample of each solution (1 mL) on the day of weight measurement (Accumet AB150 pH meter, Fisher, MA, UK). One mL of fresh solution was added to the solution to keep the volume constant, whereas for the interrupted tests, the solution was completely replaced and measured.

#### 2.2.2 Immersion Tests of AZ31 Magnesium with PF and PFS Coatings

Two PF-coated samples and one PFS sample were prepared for the experimental study. The initial PF-coated sample was submerged in a 3.5% NaCl solution, enabling a direct comparison with an uncoated AZ31 alloy specimen under identical conditions. To discriminate the effect of coating, a 5 M NaCl solution with interruption every 2 days was selected as the test medium. PF and PFS samples were ultrasonically cleaned in ethanol for 15 min and dried in air for 30 min before the test. Sample weights were measured.

The first PF sample was immersed in 100 mL of 3.5% NaCl solution, and the pH of the solution was measured every 2 days. The other PF and PFS samples were immersed in 100 mL of a 5 M NaCl solution for 14 days. For the tests in the intermittent 5 M NaCl solution, the samples were taken out every 48 h, the pH of the solutions was measured, and the discs were resuspended in fresh solution. Finally, all of the samples were ultrasonically cleaned in ethanol for 15 min and dried in air for 30 min before weighing.

### 2.3 Specimen Characterization

For metallographic characterization of cross-sections, the samples were embedded in resin and then ground with 800, 1200, and 4000 grit silicon carbide papers and finally polished with 1 and 0.25 μm oil-based diamond suspension (MetPrep, UK). Then, the samples were cleaned with ethanol in an ultrasonic bath for 15 min and dried with air.

The surface and cross-sectional morphology, along with energy dispersive analysis (EDX) spectra, were acquired using TESCAN Mira3 FEG-SEM OI (Czech Republic), focusing on representative regions to gain insights into the sample’s composition and structure. Secondary electron (SE) and backscattered electron (BSE) images of the samples were obtained at a working distance of 12 mm, an acceleration voltage of 5 kV, and a beam current of 0.8 nanoAmper. EDX images were obtained at a working distance of 15 mm, an acceleration voltage of 15 kV, and a beam current of 1 μA.

The topographic assessments of the uncoated and coated specimens were conducted using atomic force microscopy (AFM) (XE-70 model, Park Systems, Korea) before the corrosion tests. This analysis aimed to quantify the surface roughness parameters of the arithmetical mean height (*S*_a_) and maximum height (*S*_z_) of the area. Each topographic map encompassed an area of 50 × 50 μm, utilizing a grid of 512 × 512 data points.

X-ray computed tomography (XCT) imaging was also used for obtaining 3D reconstruction of the specimens after the corrosion tests. The PF-coated, PFS-coated, and the uncoated AZ31 specimens after the corrosion tests in the intermittent 5 M NaCl solution were scanned within the X-ray microscope chamber (Versa 520, Zeiss, US) at 90 V and 8 W in the flat panel mode. 1601 projections were acquired over 360° with an exposure time of 1 s per projection, and the resulting tomograms had a voxel size of nearly 30 μm. The tomograms were analyzed via Avizo 9.7 (Thermo Fisher Scientific, US).

### 2.4 Porosity and Cross-Section Thickness Analysis

Porosity analyses were performed on BSE images of the surfaces acquired before the corrosion tests for PF and PFS coatings using ImageJ software (NIH, Madison, WI, USA). The images were filtered with median filter, and then a thresholding was applied to obtain a binary image. The voids were determined by particle analysis method, and for each void, the diameter, *D*, of the equivalent circle having the same area was calculated as
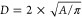
5where *A* is the area of the individual speckle. The porosity is calculated by the ratio of void area with the total area of the region of interest.

Using ImageJ software, coating thickness analysis was performed on BSE images of the cross-sectioned PF and PFS coating discs before the corrosion tests. Images were filtered with the median filter, and then a thresholding was applied to obtain a binary image of the coating before the thickness was quantified using Python’s library.^26^

## 3 Results

### 3.1 Materials Characterization Prior to Corrosion Tests

**Figure 1:**
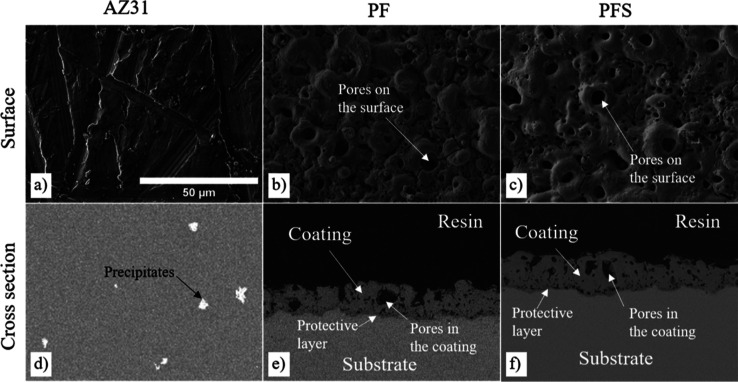
Initial surface and cross-section of the specimens before corrosion tests. (a) Uncoated AZ31 Mg alloy, (b) PF coating, (c) PFS coating, and the cross-sections of (d) uncoated AZ31 Mg alloy, (e) PF coating, and (f) PFS coating.

The surface roughness parameters of the samples are listed in Table 1. The surface of the uncoated AZ31 is quite coarse, with random scratches (Figure 1a). The cross-section of the AZ31 alloy shows that the AZ31 Mg alloy includes several randomly distributed coarse Mg_17_Al_12_ precipitates across the solid solution α-Mg (Figure 1d).^27^

**Table 1:** Surface Roughness Parameters (*S*_a_ and *S*_z_) of Uncoated, PF-Coated, and PFS-Coated Samples

	uncoated sample (μm)	PF-coated sample (μm)	PFS-coated sample (μm)
*S*_a_	0.383	1.01	0.6992
*S*_z_	1.782	7.35	5.17

The surfaces and cross-section SEM images of the coated samples before testing are shown in Figure 1b,c,e,f, respectively. The surface porosities of PF and PFS coatings are 13 and 16%, respectively, with an average pore size diameter, for both coatings, of 2.2 μm. The coated samples had cracks and pores on the sample surfaces and in the coating. The SEM images in Figure 1b,c show that the pores are surrounded by a rim of coating material that is protruding from the coating surface. The cross-section BSE images show that the average coating thicknesses for PF and PFS are 11.6 ± 2.3 and 15.7 ± 4.1 μm, respectively. The pores and cracks seen on the surface penetrate into the coating but never contact the surface of the alloy. There is a denser non-porous coating layer which measures less than 1 μm adjacent to the alloy surface.

### 3.2 Immersion Tests

**Figure 2:**
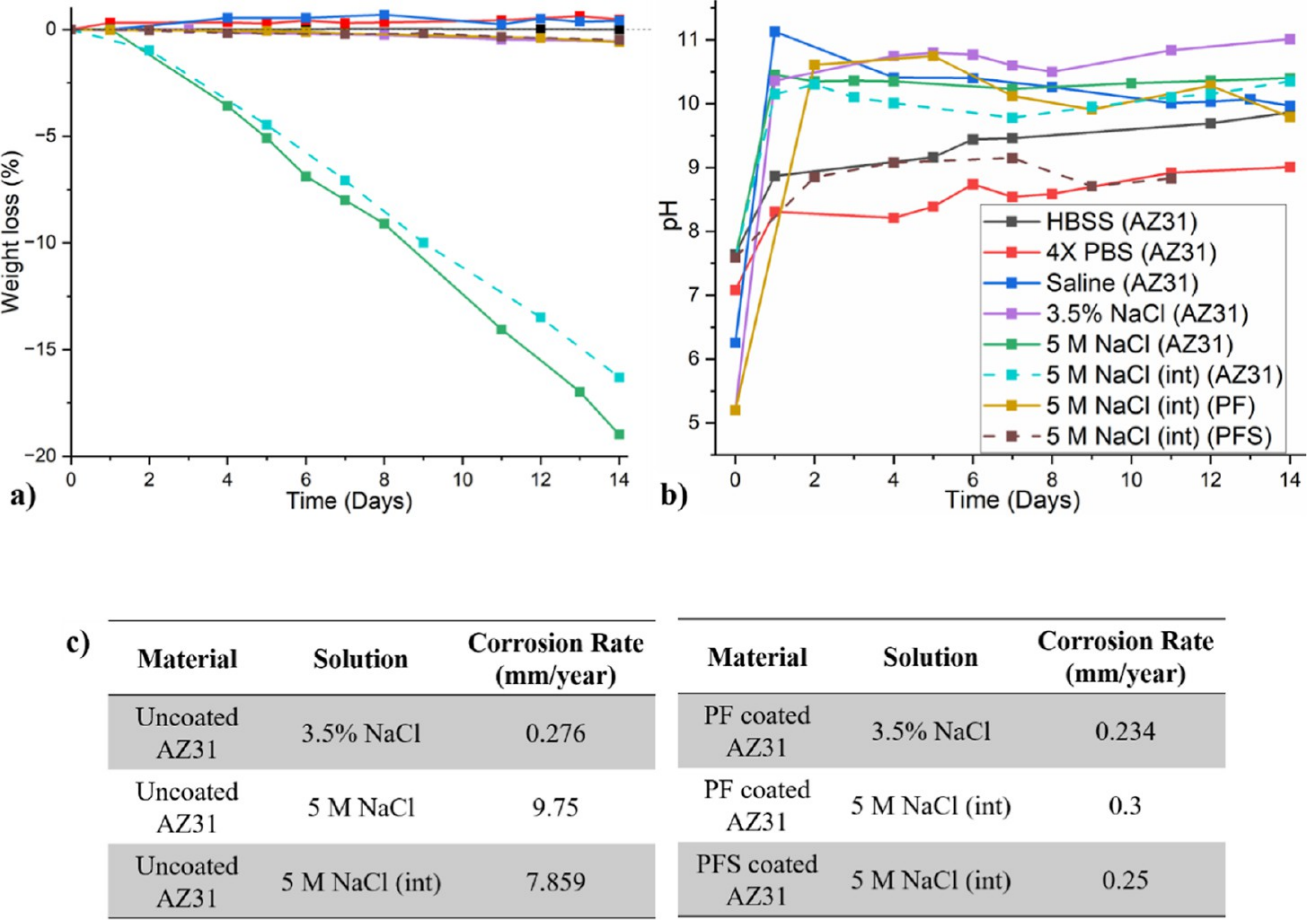
Time-dependent (a) weight loss (%) and (b) pH changes for AZ31 alloy disks in various solutions and the impact of PF coating and PFS coating on pH change in 5 M NaCl solution. Int. refers to intermittent changes of 5 M NaCl solution. (c) Corrosion rates of uncoated AZ31 Mg alloy disks after 14 days in various NaCl solutions and the effect of the PF coating and PFS coating on corrosion resistance in 5 M NaCl solution.

The corrosion behavior of the AZ31 Mg alloy was investigated through immersion in various solutions, and the resulting weight loss (%) is presented in Figure 2a. No discernible weight loss was evident after a 14-day exposure to physiological solutions, namely HBSS, PBS, 4× PBS, and saline solutions; in fact, the samples exhibited weight gain in these environments. In chloride-rich solutions, a notable weight loss was observed. Notably, the weight loss of the AZ31 samples exhibited a consistent linear decrease over time, regardless of whether the NaCl solution underwent complete refreshment or not. The coated samples displayed a slight decrease in weight.

The corrosion rates were determined based on the mass loss (Figure 2c). The results showed that no significant changes in corrosion rate were observed after 14 days in HBSS, PBS, 4× PBS, and saline solutions. However, the corrosion rate of AZ31 in 3.5% NaCl was 0.27 mm/year. In intermittent and continuous 5 M NaCl, the corrosion rates were 7.8 and 9.7 mm/year, respectively.

The corrosion rate of PF-coated samples in an intermittent 5 M NaCl solution was found to be remarkably similar to that of the PFS-coated sample when immersed in the same solution, with both exhibiting a corrosion rate of approximately 0.30 and 0.25 mm/year, respectively. Both coated samples demonstrated an exceptionally low corrosion rate after 14 days. Notably, this corrosion rate is more than 30 times lower than the corrosion rate observed in the uncoated AZ31 Mg sample under identical test conditions.

The changes in pH depending on the solution type over time are given in Figure 2b for the coated and uncoated AZ31 disks. For the continuous tests, pH values significantly increase on the first day and then slightly increase. For HBSS and 4× PBS solutions, the pH remained less than 10 during the tests; among all solutions, 4× PBS maintained the lowest pH throughout the test. However, when AZ31 was immersed in solutions containing different concentrations of NaCl, the pH rapidly increased above 10 on the first day and then remained relatively stable. The pH values of the AZ31 coated with PF coating in a 3.5% NaCl solution were similar to that of the AZ31 sample when immersed in the same solution, whereas the AZ31 Mg alloys coated with PFS coating sample subjected to an intermittent 5 M NaCl solution test demonstrated a lower pH of 9.0, which remained stable over 14 days.

### 3.3 Corrosion of Uncoated AZ31 Mg Alloy

**Figure 3:**
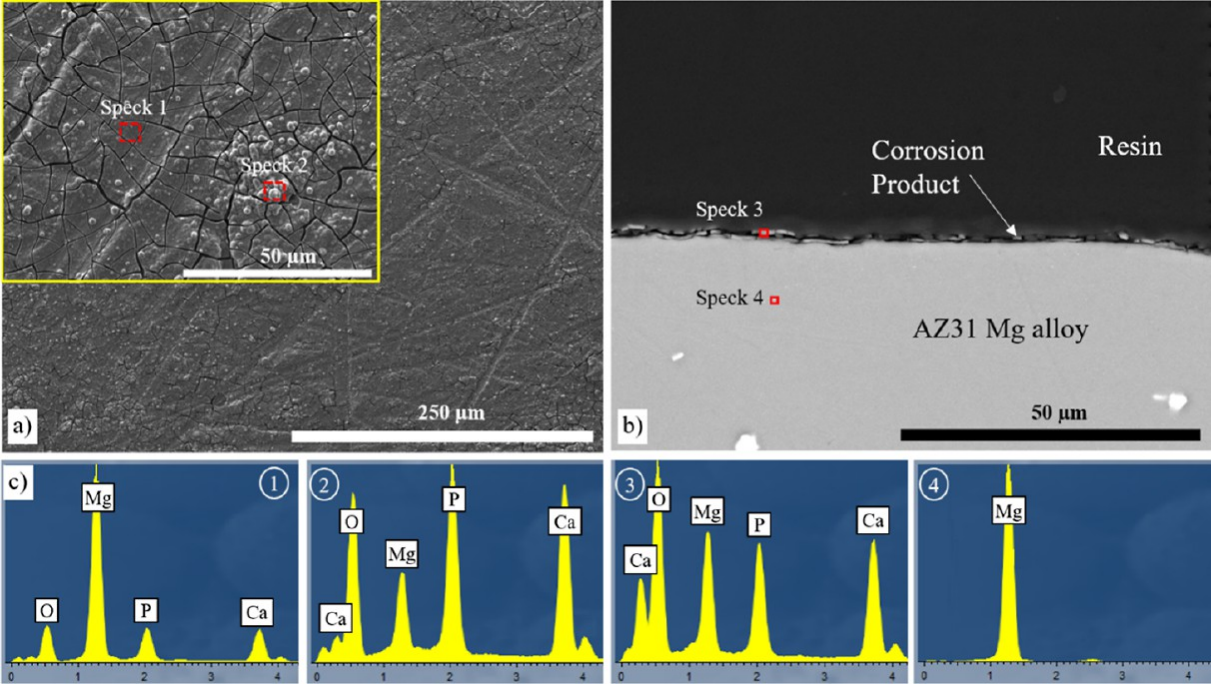
(a) Surface and (b) cross-section SEM images of the corroded uncoated AZ31 Mg alloy in HBSS solution after 14 days and (c) spectrum analysis of the selected points.

The surface and cross-section SEM images of AZ31 Mg alloys immersed in HBSS show that the cracking of the layer of corrosion products divided the surface into a network structure due to the corrosion products drying out (Figure 3a,b). The corresponding spectrum analysis in different regions reveals that high amounts of oxygen (O), phosphorus (P), and calcium (Ca) were present on the corroded surface (Figure 3c). Spectrum 1 shows aluminum (Al), O, and Ca along with Mg. Spectrum 2 corresponds to the spherical particles observed at the surface of the alloy and shows that they contain a high amount of O, P, and Ca. The peaks in spectrum 4 perfectly match the AZ31 Mg alloy composition.

**Figure 4:**
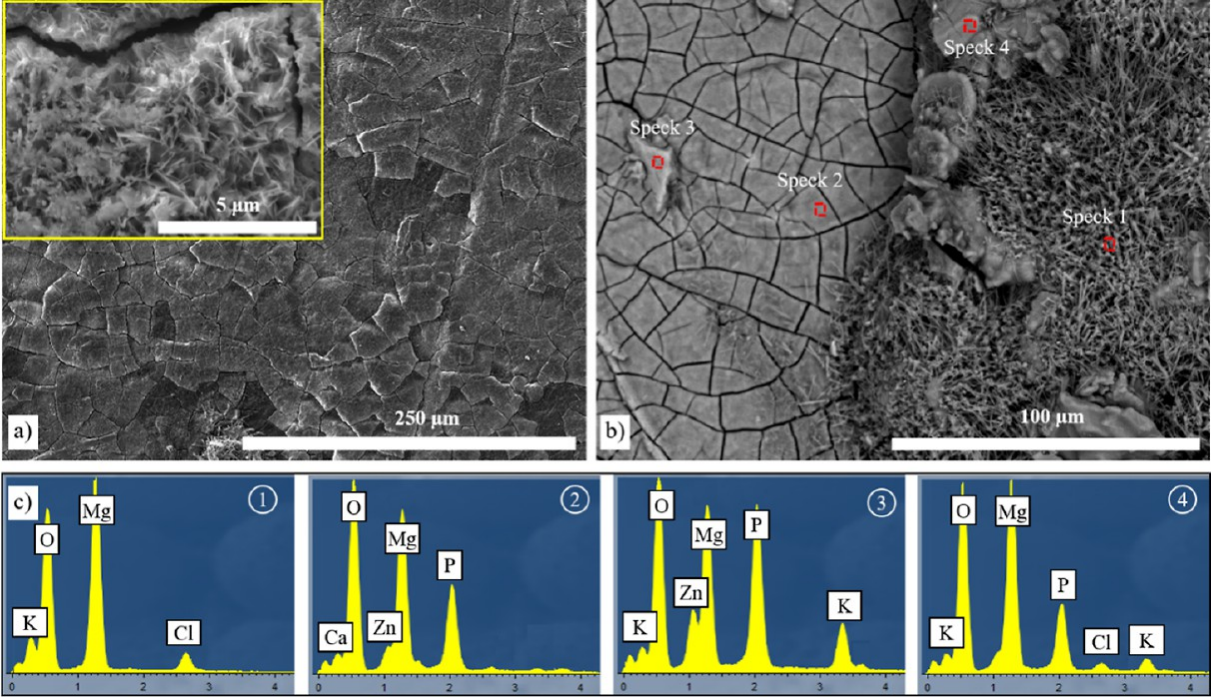
Surface (a) SE SEM and (b) EDX images of the corroded uncoated AZ31 Mg alloy in 1× PBS after 14 days and (c) spectrum analysis of the selected points.

**Figure 5:**
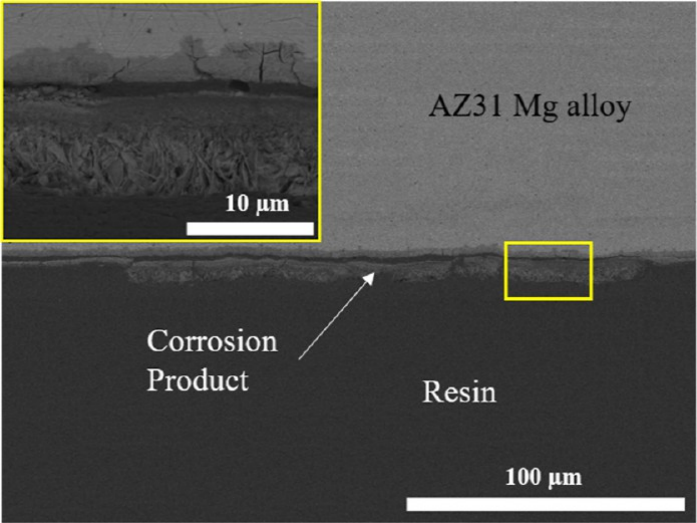
Cross-section SEM images of the corroded uncoated AZ31 Mg alloy in 4× PBS after 14 days show the detachment between a corrosion layer at the surface and a corrosion layer adjacent to the alloy surface.

The microstructure of the surface of the AZ31 Mg alloy in 1× PBS and 4× PBS solutions after 14 days shows that a precipitation consisting of sodium (Na), potassium (K), and P covers the alloy surface (Figure 4). The corrosion products are cracked and create a network structure (Figure 4a). Most of the surface is covered by needle-shaped corrosion products with diameters of less than 1 μm. In some regions, larger precipitates with a spoke-like structure can be seen. The chemical elemental composition of the spoke-like structure is composed of K, O, Na, P, and Mg, whereas that of the needle-shaped products have less K. Cross-sections revealed a double corrosion layer (Figure 5).

**Figure 6:**
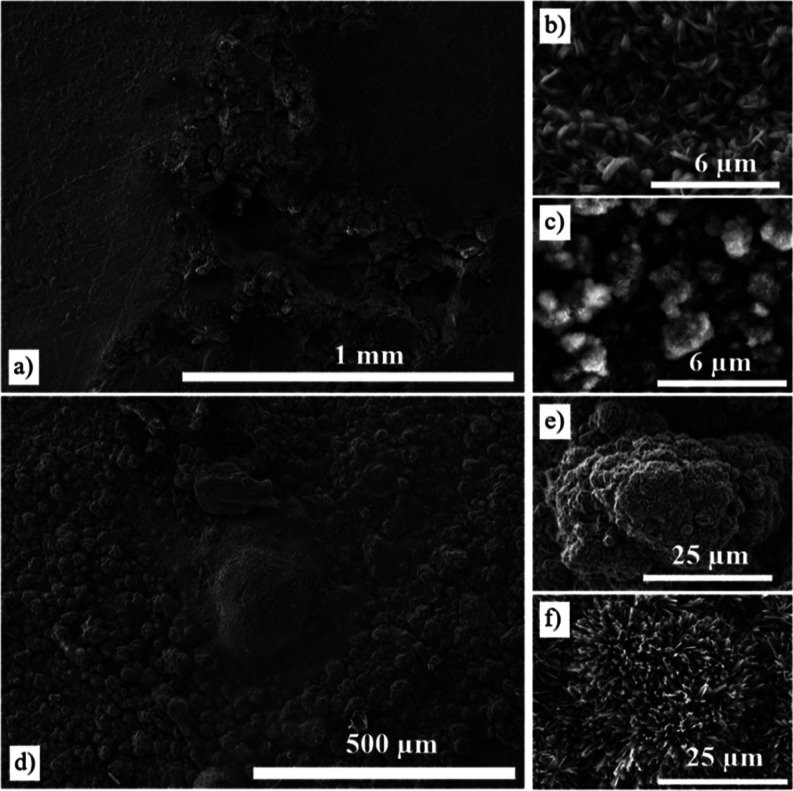
Surface SEM images of the corroded uncoated AZ31 Mg alloy in saline (a–c) and 3.5% NaCl solutions (d–f) after 14 days.

The corrosion morphologies of the AZ31 in saline and 3.5% NaCl solutions after 14 days show that surfaces are entirely covered by a uniform layer of corrosion product (Figure 6).^28^ The scratches remained from the manufacturing are visible in the sample immersed in saline solution (Figure 6a), whereas corrosion products are formed on the surface of the sample immersed in 3.5% NaCl solution (Figure 6d). Microstructural examination shows that the corrosion structures are morphologically heterogeneous with leaf-like (Figure 6b), globular type (Figure 6c–e),^29^ and needle-like clusters (Figure 6e).^30^ Needle-like clusters are formed perpendicular to the surface on the corrosion layer in a 3.5% solution.

**Figure 7:**
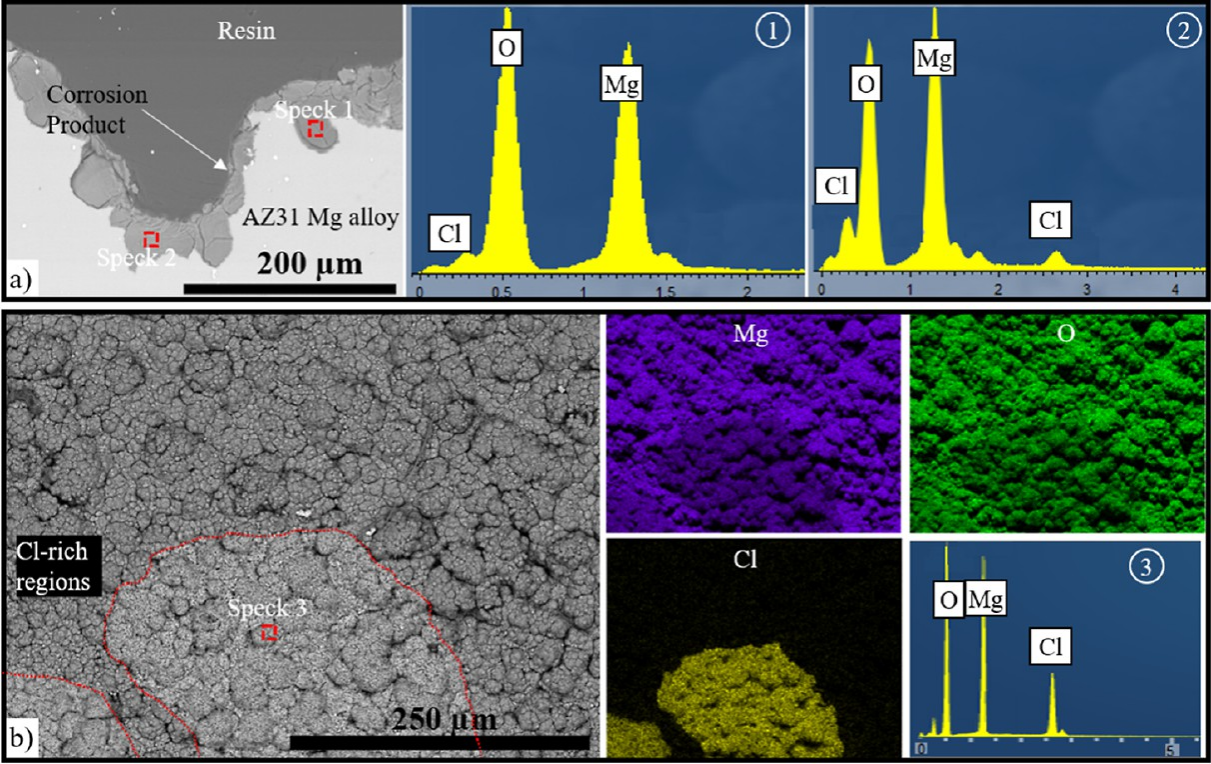
(a) Cross-section BSE image of the corroded uncoated AZ31 Mg alloy in saline solution after 14 days and the spectrum analysis of the selected points. (b) Surface BSE image of the corroded AZ31 Mg alloy in 3.5% NaCl solution after 14 days and mapping and the spectrum analysis of the selected points. Cl-rich regions are shown with dotted red lines.

The corrosion products of the samples are similar to each other. SEM analysis of the cross-section of the AZ31 Mg sample in saline solution shows that a thick and irregular corrosion layer forms on the surface (Figure 7a). EDX showed that the corrosion product seen as a layer on the surface of the alloy and in pits was composed of Mg oxides and hydroxides, and in some regions, they were also Cl-rich (Figure 7a—spectrum 1 and 2).^31^ The chemical composition of the surface of the sample after the corrosion test in 3.5% NaCl solution, obtained by EDX, shows that all regions are rich in oxygen, whereas the corrosion layer formed on the surface is a Cl-rich region, which is related to the presence of MgCl_2_.

**Figure 8:**
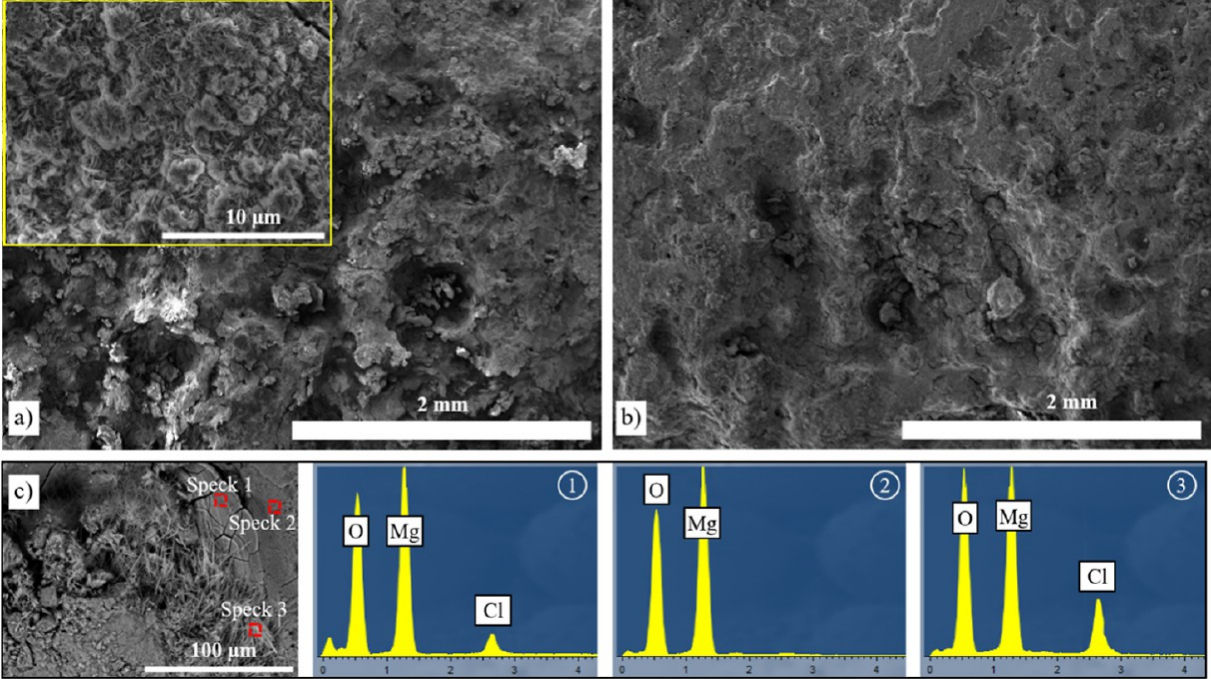
Surface SE SEM images of the corroded uncoated AZ31 Mg alloy in (a) continuous and (b) intermittent 5 M NaCl after 14 days and (c) spectrum analysis of the selected points in the surface of the BSE image taken after the intermittent test.

Continuous and intermittent 5 M NaCl solution tests showed much higher corrosion rates. During the continuous test, precipitates formed and remained in the test fluid, whereas in the intermittent test, where the solution was changed every 2 days, the level of precipitates was reduced. Severe damage was associated with the formation of corrosion products seen on the alloy surfaces (Figure 8a,b). The most common corrosion features for both tests were deep pits. In the sample immersed in an intermittent 5 M NaCl solution, the surface morphology was variable (Figure 8c), and EDS analysis at different points shows that in some regions only Mg and O exist (Figure 8c—spectrum 2), while in some other regions, Cl^–^ was also found in the corrosion layer (Figure 8c—spectrum 1 and 3).

**Figure 9:**
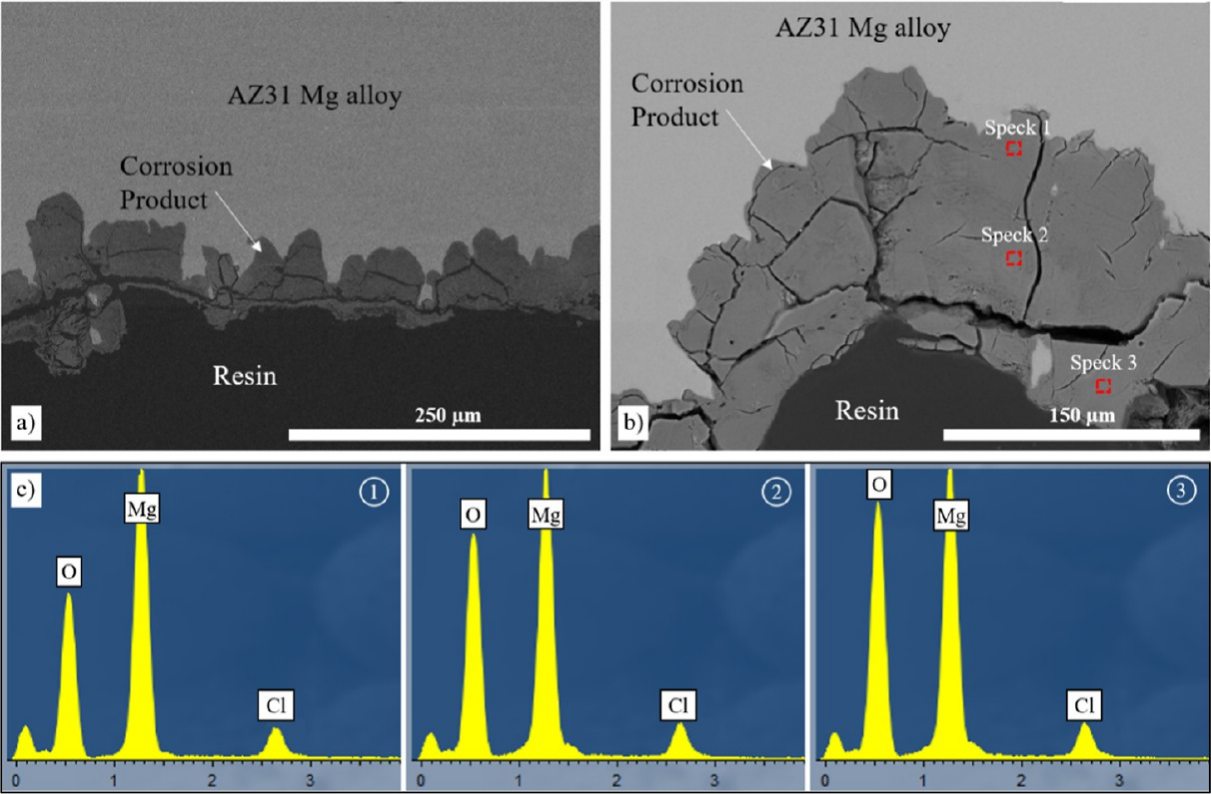
Cross-section BSE SEM images of the corroded uncoated AZ31 Mg alloy in intermittent 5 M NaCl (a,b) after 14 days and (c) spectrum analysis of the selected points in (b).

The cross-section images of samples in a 5 M NaCl solution show deep penetration of the corrosion products, and the sample edges have become uneven on a large scale (Figure 9a,b). Spectrum analysis of the corrosion products shows an increasing amount of oxygen from the inner toward the outer surface (Figure 9c). Also, there is a slight increase in Cl.

### 3.4 Corrosion of Coated AZ31 Mg Alloy

**Figure 10:**
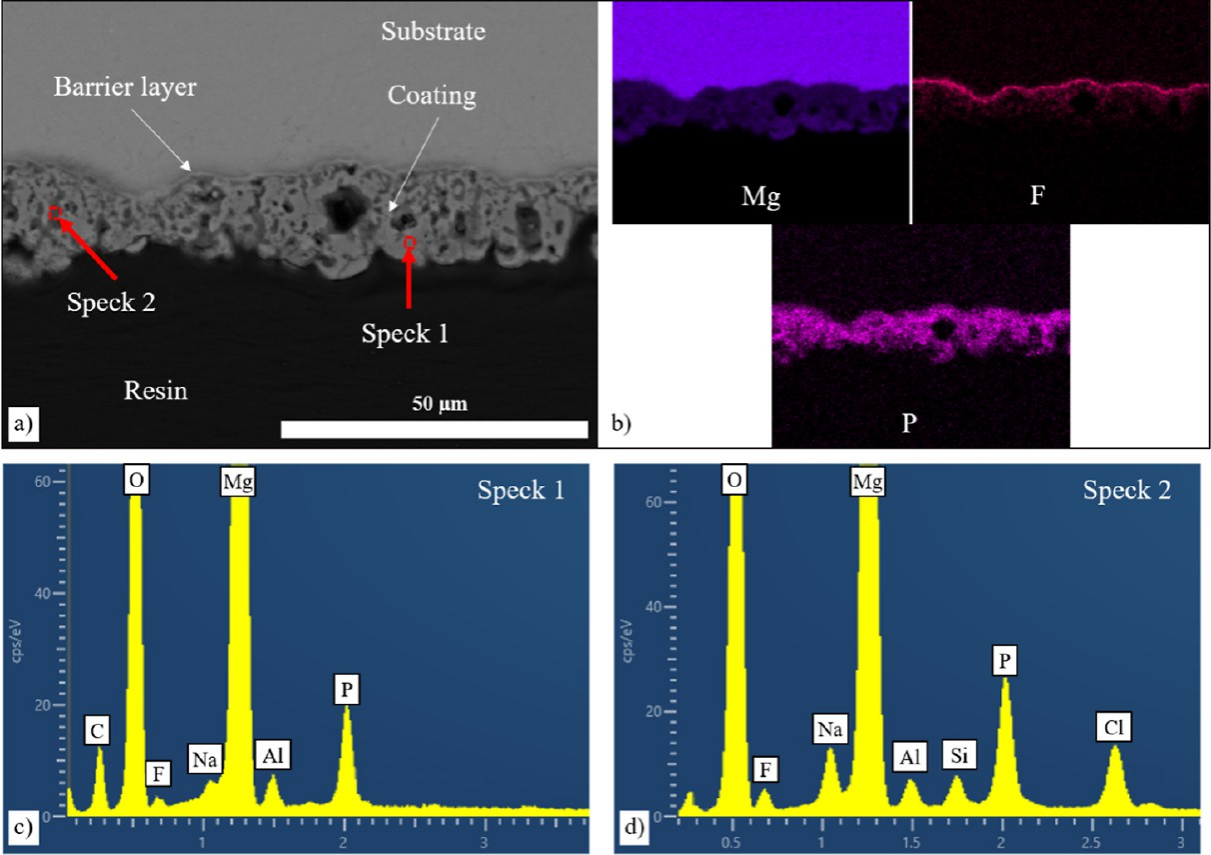
(a) SEM cross-section of the corroded AZ31 Mg alloy coated with PF coating in 3.5% NaCl solution after 14 days, (b) elemental composition corresponding to the area, and (c,d) point analysis.

In the 3.5% NaCl solution, corrosion was observed on the PF-coated AZ31 Mg disc, with the corrosion products exclusively forming on the coating without reaching the substrate (Figure 10). Notably, the coating incorporates a fluoride-rich barrier layer adjacent to the alloy surface that measures less than 1 μm in thickness and impedes the progression of corrosion. EDX analysis shows corrosion occurring in different locations on the same disc with local higher concentrations of Cl^–^ and Na^+^ ions in some regions.

**Figure 11:**
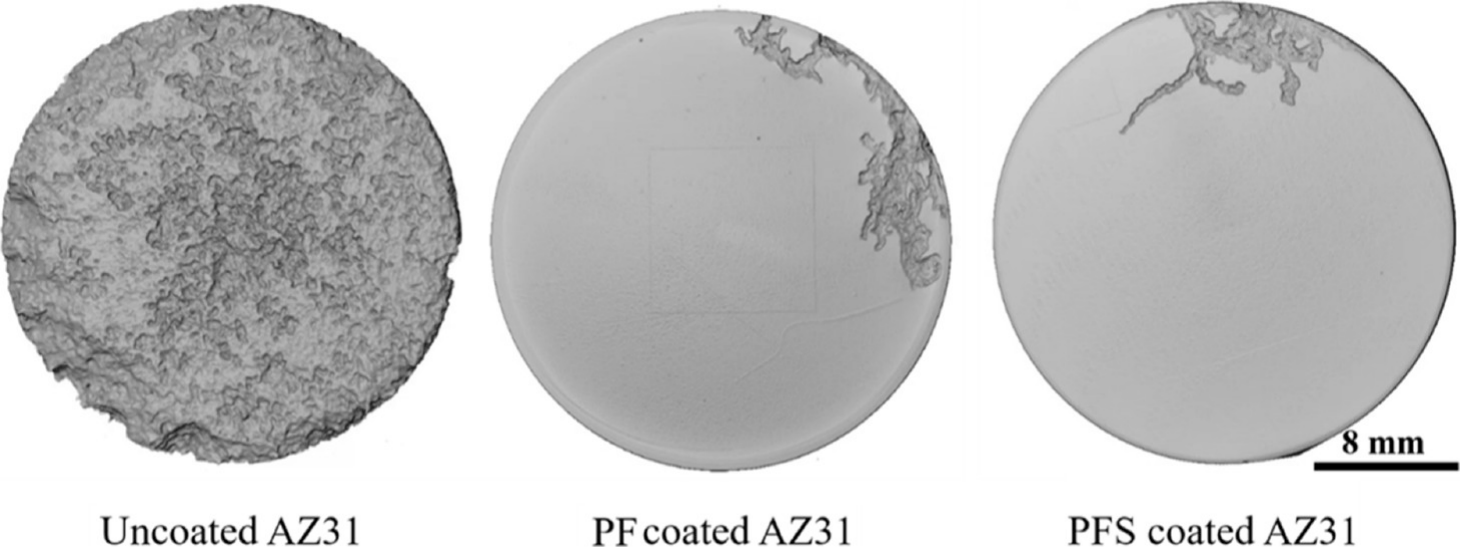
XCT reconstruction for the corroded uncoated AZ31, PF-coated AZ31, and PFS-coated AZ31 samples immersed in an intermittent 5 M NaCl solution after 14 days.

The results obtained from the XCT analysis of the corrosion surfaces of the uncoated AZ31, PF-coated AZ31, and PFS-coated AZ31 Mg discs in the intermittent 5 M NaCl solution are shown in Figure 11. The XCT reconstruction reveals distinct differences in the corrosion behavior between the three specimens. The uncoated AZ31 Mg alloy exhibits severe damage due to corrosion, with a substantial portion of its surface affected. In contrast, both the PF-coated AZ31 and PFS-coated AZ31 Mg discs display significantly reduced corrosion. Only a small fraction of their surfaces show signs of corrosion, indicating the protective capabilities of the coatings. The majority of the coatings on AZ31 discs remain attached to the substrate. However, the presence of pores in the coatings leads to solution penetration down to the barrier layer, which in places was incomplete, suggesting a time limitation in providing comprehensive protection against the aggressive solution.^22^

**Figure 12:**
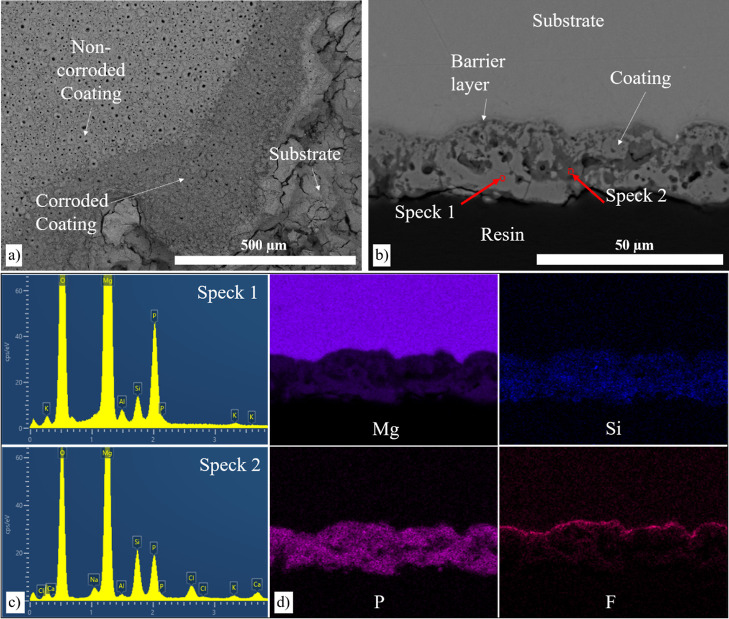
Characterization of the corroded AZ31 Mg alloy coated with PFS coating in intermittent 5 M NaCl solution after 14 days. (a) Surface, (b) cross-section of the corroded sample, (c) corresponding point analysis, and (d) elemental analysis of the cross-section.

In the 5 M NaCl solution, the microstructural investigation of the corrosion of the PFS-coated AZ31 disc showed slight deterioration (Figure 12) with some areas of the coating showing evidence of corrosion products. However, in other regions, there was no evidence of corrosion products on the coating surface. This contrasted with areas where complete dissolution of the coating was seen, resulting in severe corrosion of the underlying substrate. The cross-section analysis depicts a non-uniform corrosion pattern, with some regions displaying corrosion while others remained uncorroded (Figure 12b). In the noncorroded region (spectrum 1 in Figure 12c), a higher concentration of P ions was observed. Conversely, areas with corrosion products exhibit lower P levels but higher concentrations of Cl^–^.

## 4 Discussion

This study aims to assess the corrosion behavior and products of the AZ31 Mg alloy in various corrosion media, as well as the protective effect of two phosphate-based ECO coatings. The findings are expected to provide guidance and serve as a reference for the application of Mg alloys in physiological environments where the test can differentiate between coated and uncoated AZ31. The results demonstrate that uncoated AZ31 alloy exhibits undetectable corrosion rates when exposed to physiological solutions due to the formation of a protective corrosion layer on the surface. However, more aggressive NaCl solutions lead to increased corrosion that could be measured over a short period of time (14 days). These tests were also able to differentiate the effects of a coating on corrosion, with a corrosion rate more than 30 times lower than that of the uncoated alloy.

There are several ways to directly measure the corrosion of magnesium alloys, which include measuring the volume of hydrogen generated, dynamic polarization tests, weight loss, and volumetric change. Indirect measurements are also important and can be related to the function of the alloy in a biomedical setting. These measurements include a reduction in strength measured either monotonically or by measuring a reduction in fatigue properties. In the short term, potentiodynamic polarization tests are very valuable to understand the corrosion of the alloys and indicate the potential of the coatings to prevent corrosion, but these are difficult to carry out in a longitudinal study over 2 weeks, as the coatings as well as the alloy surfaces, will be subject to corrosion.

In HBSS solution, the AZ31 first forms the Mg(OH)_2_ layer which undergoes a reaction to form MgCl_2_ in aqueous chloride solutions. Then, the compound slowly dissolves into the solution. The corrosion product appears to show a cracked surface, which is attributed to the drying of the surface.^32^ Simultaneously, the calcium and phosphorus ions present in HBSS undergo precipitation and are deposited on the surface of the sample, forming a layer of precipitates (Figure 3a). Those precipitates, possibly magnesium phosphate (Mg_3_(PO_4_)_2_) and hydroxyapatite (Ca_10_(PO_4_)_6_(OH)_2_), are produced on the Mg(OH)_2_/CaP film due to the reaction between PO_4_^3–^ with Ca^2+^ and Mg^2+^ (Figure 3c—Speck 2).^32^ The AZ31 Mg alloy immersed in HBSS and NaCl solution increases the pH due to releasing OH^–^ ions as a result of the cathodic reaction and forms precipitations of MgO and/or Mg(OH)_2_ products.

The osteoconductive property of biomaterials can be measured by determining the bioactivity of the surface, which is related to build up the calcium phosphate apatite layer on the surface of the biomaterial in a medium that is similar in composition to inorganic body fluids. The faster the buildup of the apatite layer and the closer the calcium–phosphate ratio of the apatite is to hydroxyapatite, the more osteoconductive the material. The hydroxyapatite layer is a natural scaffold for cells, stimulating them to adhere and multiply.^33,34^ Hydroxyapatite [Ca_10_(OH)_2_(PO_4_)_6_)] is calcium orthophosphate, a salt of the tribasic acid orthophosphoric H_3_PO_4_, with a Ca/P molar ratio equal to 1.667. In order to recreate the tissue environment under laboratory conditions, fluids that simulate the inorganic constituents of body fluids, such as HBSS, are used. HBSS contains inorganic components of blood plasma, rich in chloride ions, which in the tissue environment are mainly responsible for the corrosive processes of metallic biomaterials. The chemical composition of HBSS was selected so that the concentration of ions was comparable to the concentration of biological fluids, and the pH value was the same as that of human blood. Osteoconductive biomaterials, when immersed in HBSS, have been shown to form an apatite layer on their surface.^35^ So, the accumulation of magnesium phosphate (Mg_3_(PO_4_)_2_) and hydroxyapatite Ca_10_(OH)_2_(PO_4_)_6_) on AZ31 may indicate that the corrosion products derived from AZ31 would provide an alloy surface that has bioactive properties and is potentially osteoconductive. Regarding the calcium–phosphate ratio, the calculated value is 1.33, implying a slight variance from the Ca/P molar ratio found in hydroxyapatite. Valuable information on the bioactivity of these materials can be obtained by using physiological inorganic fluids; however, in our study the rate of corrosion in HBSS was low, and the environment was not considered aggressive enough to measure the differences in coated and uncoated alloys. The corrosion rate for the alloy in 1× and 4× PBS was difficult to measure, and all specimens, even after 14 days of immersion, gained weight due to the accumulation of corrosion products on the surface of the alloy.

In the case of AZ31 samples immersed in PBS, the presence of phosphate ions (HPO_4_^2–^ and H_2_PO_4_^–^) can trap the OH^–^ ions, preventing the formation of highly alkaline pH conditions that promote the precipitation of Mg(OH)_2_.^18^ In contrast, phosphate ions react with magnesium ions (Mg^2+^) to create water-insoluble magnesium phosphate (Mg_3_(PO_4_)_2_), which then accumulates on the surface and results in slower dissolution of the Mg alloy.^19^

The cross-sectional analysis of the AZ31 sample corroded in a PBS solution shows the presence of two layers (Figure 5). One layer is in direct contact with the material due to the cathodic reaction, and the layer on the surface is associated with the accumulation of corrosion products. This layer facilitates a diffusion-controlled corrosion process, resulting in a decrease in the corrosion rate. Kim et al.^11^ explained that stable Mg(OH)_2_ creates favourable conditions that nucleate and grow further corrosion products on its surface that remain stable over an extended period. The growth of the corrosion product layer mainly composed of MgO and PO_4_^3–^ is responsible for slowing down the corrosion reaction, thereby increasing the corrosion resistance.^18^ After a certain period of immersion in PBS, the solid magnesium phosphate layer can be dissolved due to the accumulation of chloride ions and the expansion of corrosion pits.^19^

The corrosion tests performed at three different NaCl concentrations (0.9%, 3.5%, and 5 M) indicate the effect of Cl^–^ concentration on the corrosion rate and surface degradation of the alloy. In the first test with a 0.9% NaCl solution, the observed corrosion filaments and pits (Figure 6) indicated that even at a relatively low concentration, corrosion is still present. Cross-sections show pitting of the alloy with Mg and oxygen detected, indicating the formation of an adherent Mg hydroxide layer. The second test utilizing a 3.5% NaCl solution results in similar morphology, but overall there is greater and deeper pitting compared to that in the 0.9% NaCl test.^28^ This aligns with expectations, as higher Cl^–^ concentrations typically accelerate the corrosion process. The increased severity of corrosion in the 3.5% NaCl solution demonstrates the sensitivity of the AZ31 Mg alloy to higher chloride concentrations.

The 5 M NaCl solutions induced severe corrosion due to the higher concentration of corrosive ions. The test with the same solution for 2 weeks resulted in greater corrosion compared to the test where the solution was changed every 2 days. This indicates that the corrosion is intense and leads to the breakdown of the surface layer and corrosion products. Conversely, the samples in the tests where the solution was changed every 2 days exhibited slightly better corrosion resistance. Although corrosion still occurred and resulted in deep penetration, there are regions that retained the initial shape of the sample. This suggests that the periodic change of the solution helped mitigate the corrosion process to some extent, preserving certain regions of the sample’s surface. The Cl^–^-containing solution promotes fast corrosion of AZ31 and forms Mg(OH)_2_ at the beginning of the test as a protective layer; however, the surface layer is not fully protective since it has several cracks and a flaky appearance.^30^

The underlying reason for the significant variation in the form of the corrosion products containing Cl^–^ is not well understood, although it is possible that the corrosion may be influenced by the substrate’s texture and grain size.^11^ Secondary-phase corrosion can lead to an increase in pitting associated with areas where the corrosion product layer is prone to breaking, resulting in more intense corrosion processes. In the AZ31 Mg alloy, corrosion can start near the intermetallic phase, which acts as a galvanic cathode and accelerates the corrosion of the alpha phase.^30,36^ The presence of gaps or interruptions in the layer of corrosion products encourages continuous exposure of the underlying metal. This exposure can lead to the formation of new crystalline structures, which often take the shape of needle-like or flower-like crystals.^30^ In this study, the observed crystallization phenomenon was found in the specimens of AZ31 alloy immersed in all solutions, except for HBSS solution.

Studies have shown that the degradation rate of Mg could be controlled by surface modification technologies such as chemical conversion, hydrothermal treatment,^37^ sol–gel method,^38^ polymer coatings, plasma spraying, microarc oxidation,^39^ magnetron sputtering, and electrochemical deposition.^40^ The electrochemical deposition method has the ability to produce coatings on the surface of samples with different shapes at relatively low temperatures. This technique can also produce coatings containing calcium and phosphate that are biocompatible and that may be bioactive.^41^ The morphology and composition of the coating can be controlled by adjusting the parameters of the electrochemical deposition, and this technique allows complex 3D surfaces to be coated.^42,43^

Two different compositional electrolytes were used in this study. One employs phosphate and fluoride (PF) salts and the other phosphate, fluoride, and silicate (PFS) salts. These two electrolytes have been selected from a variety of candidate electrolyte solutions investigated for building compact nanocrystalline ceramic adherent layers on magnesium alloys. None of those constituent elements had a critical effect on the plasma discharge pattern and characteristics. The required soft sparking effect has been achieved by selection of the forming electrical pulse parameters (as described above).

It would have been useful to measure the evolution of hydrogen, which on the uncoated alloy occurs rapidly and then slows,^44^ whereas the corrosion rate as measured by weight loss for the uncoated alloy is relatively constant over the duration of the experiment. Hydrogen evolution is a consequence of hydrolysis and the release of hydroxide ions. In this study, we measured the pH increase associated with AZ31 in different solutions. The pH increased rapidly and then remained at a constant level. Interestingly, the pH of the solutions with AZ31 samples with lower chloride ions showed a similar rapid increase, to the 5M concentrations, but the pH reading plateaued at lower levels.

The similarity in corrosion rates of the coated samples when incubated in high chlorine concentrations makes it challenging to differentiate their corrosion behavior based solely on corrosion rate measurements, and both coated alloys offer superior corrosion resistance when exposed to highly concentrated chlorine ions. This indicated the importance of calculating corrosion rates while observing the morphology of the surfaces. The corrosion rates of the PF coating and AZ31 alloy in 3.5% NaCl solution are found to be similar at 0.23 and 0.276 mm/year, respectively. This similarity in corrosion rates makes it challenging to differentiate their corrosion behavior. However, microstructural investigation shows that the corrosion products are generally formed on and in the voids of the coating, which reduces in thickness, and the alloy surface is protected from the corrosion process. This phenomenon was accompanied by an increase in porosity after 2 weeks of exposure. Both the coatings investigated have a dense nonporous thin layer, rich in fluorine, that may act as a barrier adjacent to the implant surface.^45^

These findings suggest that while the corrosion rates of the PF coated and AZ31 alloy in 3.5% NaCl solution may appear similar, their corrosion mechanisms and behavior at the microscale differ significantly. The formation of corrosion products on the surface of the PF coating indicates a protective effect of the coating, preventing substantial material removal.

**Figure 13:**
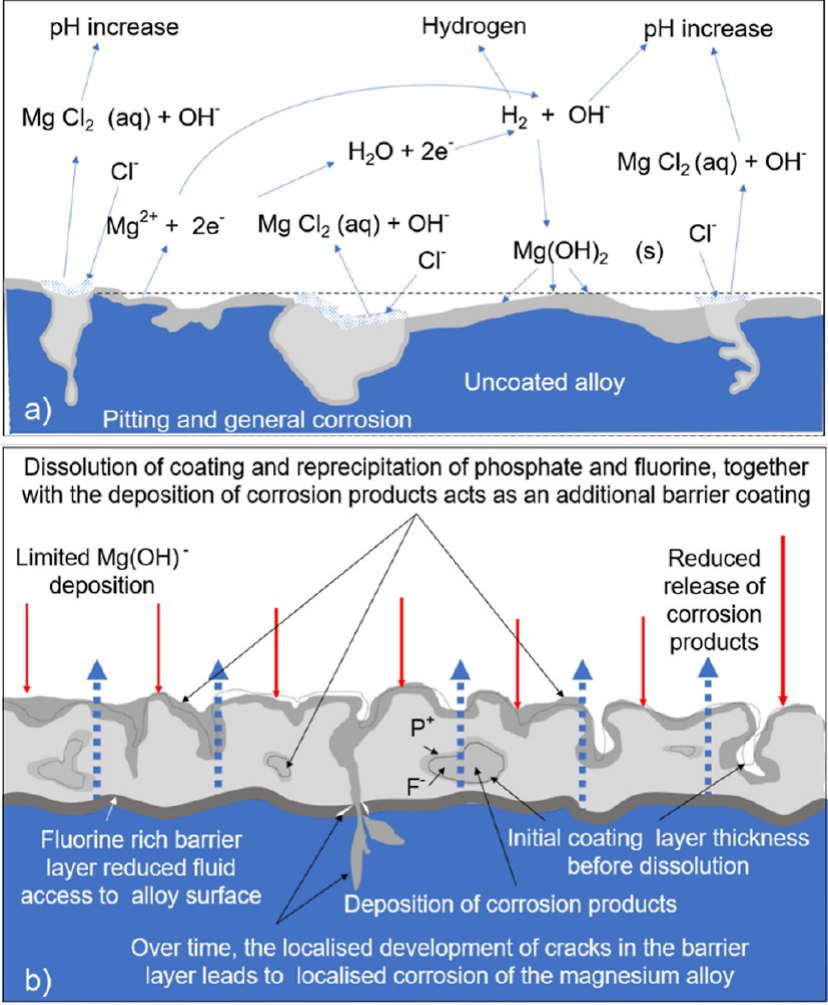
Schematic of (a) corrosion mechanisms in an uncoated magnesium alloy and (b) corrosion protection mechanisms by PF- and PFS-coated magnesium alloy.

The corrosion rates of PF- and PFS-coated AZ31 in a 5 M NaCl solution highlight significant differences in their corrosion behavior. This disparity in corrosion rates allows for a straightforward comparison between the coated and uncoated specimens. The porous structure of the coated samples transforms into a corroded surface, with complete removal of the coating observed in certain regions, observable on a macroscale.^45^ However, in most areas, the coating remains. Cross-sectional images reveal degradation of the coating at specific points, resulting in a decrease in the coating thickness (Figure 12b). Furthermore, corrosion and the associated deposition of corrosion products also occur within the coating. Analysis of BSE images of PFS coating reveals that the corrosion formation does not penetrate the Mg alloy over most of the surface but rather affects the coating. In comparison, the surface of AZ31 in a 5 M NaCl solution is severely pitted over its entire surface. Figure 13 illustrates the schematic of the degradation process of an uncoated magnesium alloy and the protective corrosion mechanisms conferred by PF and PFS coatings in a NaCl solution.

This finding highlights the potential benefits of PF and PFS coatings in chloride-containing environments. However, in order to enhance the corrosion resistance of Mg alloys further, it is imperative to undertake comprehensive investigations that concentrate on optimizing the properties and structure of the coatings.

## 5 Conclusions

The present study investigated the corrosion behavior of the AZ31 Mg alloy in various corrosion media, and the effects of different coatings on corrosion resistance were examined. The results show that the uncoated AZ31 alloy exhibits low corrosion rates in physiological solutions due to the formation of corrosion products, leading to a protective layer on the sample surfaces. The corrosion products formed on the surfaces of the corroded samples consist of various compounds, such as phosphates, oxides, hydroxides, and calcium. However, upon exposure to more aggressive NaCl solutions, the corrosion rates increase significantly.

Intermittent 5 M NaCl solution proves to be a favorable medium for accelerating corrosion tests, providing information on the corrosion resistance in chloride solutions such as body fluid. Lower concentrations of NaCl still induce corrosion, but higher concentrations of NaCl result in more severe corrosion effects. In this accelerated test, PF and PFS coatings demonstrate improved corrosion resistance compared to the uncoated AZ31 alloy. Although some deterioration of the coating is observed, it protects the substrate. Controlled corrosion of the Mg alloys is important, as initially the alloy has to provide enough strength to resist the imposed load. The work presented discerns the effect of coatings on the corrosion of magnesium alloys in a short-term in vitro test, so that coatings can be taken forward and used in a biological environment. The chloride ion concentrations are much higher than in a biological environment, and it remains to be seen if the protective mechanisms proposed in this paper actually are the same processes that protect the alloy in an in vivo situation. Coating will help control the dissolution of the alloy and preserve the load-bearing capacity of the material. The optimal rate of corrosion will depend on the biological environment. The coatings in this work may provide a suitable way of optimizing the inhibition or delay in corrosion of Mg alloys. In this study, we carried out extensive morphological analysis of the corrosion product on uncoated and coated alloy, and we combined this with elemental analysis. This can provide only limited information on the composition of the corrosion products. Although this was not the main aim of this study, it would have been useful to better understand the configuration of the corrosion products under different testing conditions. In the future, X-ray diffraction before and after testing could provide further information on the composition of the corrosion products on coatings and uncoated alloys.
